# A New Non-Destructive TDR System Combined with a Piezoelectric Stack for Measuring Properties of Geomaterials

**DOI:** 10.3390/ma9060439

**Published:** 2016-06-02

**Authors:** Chanyong Choi, Minwoo Song, Daehyeon Kim, Xiong Yu

**Affiliations:** 1 High Speed Railway Research Division, Korea Railroad Research Institute, 176, Cheoldobangmulgwan-ro, Uiwang-si, Gyeonggi-do 437-757, Korea; cychoi@krri.re.kr; 2 Department of Civil Engineering, College of Engineering, Chosun University, 375 Seosuk-dong, Dong-gu, Gwangju 501-759, Korea; sweet_smw@naver.com; 3 Department of Civil Engineering, College of Engineering, Case West Reserve University, Cleveland, OH 44106, USA; xiong.yu@case.edu

**Keywords:** TDR, dry density, water content, elastic modulus, soil, compaction

## Abstract

Dry density and water content are two important factors affecting the degree of soil compaction. Conventional methods such as the sand cone test and the plate load test are used to measure such properties for evaluating the degree of compaction and the stiffness of soil in the field. However, these tests are generally very time-consuming and are inherent with some errors depending on the operator (in particular for the sand cone test). Elastic modulus is an indicator to describe the stress-strain behavior of soil and in some cases is used as a design input parameter. Although a rod type TDR (Time Domain Reflectometry) system has been recently proposed to overcome some shortcomings of the conventional methods (particularly the sand cone test), it requires driving the probes into the ground, thus implying that it is still a time-consuming and destructive testing method. This study aims to develop a new non-destructive TDR system that can rapidly measure the dry density, water content, and elastic modulus of soil on the surface of compacted soil, without disturbing the ground. In this study, the Piezoelectric Stack, which is an instrument for measuring the elastic modulus of soil, has been added to the TDR system with a flat type probe, leading to a non-destructive TDR system that is capable of measuring the dry density, water content, and elastic modulus of soil. The new TDR system developed is light enough for an engineer to carry. Results of the standard compaction and TDR tests on sand showed that the dry densities and the moisture contents measured with the new TDR system were in good agreement with those measured with the standard compaction test, respectively. Consequently, it appears that the new TDR system developed will be very useful to advance the current practice of compaction quality control.

## 1. Introduction

For most of the civil engineering projects that are underway to construct structures and facilities, the first task is to firmly compact the soil on which they will rest, so that they can serve their planned functions appropriately. Compaction of the soil is, in a sense, the most important work for ensuring a structure’s durability. The degree of compaction (*i.e.*, well-compacted or poorly compacted) is typically determined by the water content and the dry density.

In order to obtain these properties, both the field density test (*i.e.*, the sand cone test) and the plate load test, which have low reliability due to human error and long testing time, have still been employed in Korea. However, these conventional tests in compaction quality control are not only time-consuming but also cost-ineffective. Note that compaction quality control is very important, as non-uniform compaction quality in civil engineering works may result in various problems such as decreased bearing capacity, non-uniform settlement of subgrade, and cracks in the structures.

To address the aforementioned issues, a number of researchers in the US and European countries have actively studied methods of estimating the dry density and water content related to subgrade compaction quality, and the TDR (Time Domain Reflectometry) technology used in this study has been increasingly utilized for this purpose.

A lot of research on TDR has been done since it was introduced to locate discontinuities of a transmission line in the power and communication industry in the 1950s. Hugo Fellner Fedegg [1] used this technology to measure the dielectric permittivity of liquids, leading to an increased use of TDR. Topp *et al.* [2] showed that the dielectric permittivity of soils is highly dependent upon the water content of the soil, and proposed a model for measuring the soil volumetric water content valid for a wide range of soils. Roth *et al.* [3] and Dirksen *et al.* [4] argued that the model proposed by Topp *et al.* [2] is not applicable for organic soils and clays as significant errors may occur in the relationship between the dielectric permittivity and the water content.

Malicki *et al.* [5] subsequently suggested an equation accounting for the effect of soil density. However, this equation suggested by Malicki *et al.* [5] is represented with the volumetric water content, while the gravimetric water content is used by geotechnical engineers, and the calibration equation including the density effect of soil is very complex and difficult to use. A linear calibration equation containing the gravimetric water content was suggested by Siddiqui *et al.* [6], which reveals satisfactory results for a variety of soils [7].

Yu *et al.* [8] suggested a calibration equation for measuring the dry density and gravimetric water content of soil by using dielectric permittivity and total electrical conductivity. This equation has been well recognized as a reliable calibration equation for measuring the dry density and gravimetric water content of the soil. Thring *et al.* [9] recently proposed three relatively simple methods to estimate the gravimetric water content from volumetric water content (derived from the measured apparent relative dielectric permittivity values) and dry density depending on the amount of information known about the soil.

The ASTM TDR technique, developed by Professor Drnevich and his colleagues of Purdue University, the USA, has inspired researchers to study the TDR for *in-situ*-measuring the dry density and water content of soils. In addition, Ochsner *et al.* [10] measured the degree of saturation and bulk density by using a Thermo-TDR technique. Liu *et al.* [11,12] developed a Thermo-TDR sensor based on the results of Ren *et al.* [13] and measured both the physical properties of soil and the heat property.

However, as this technique requires inserting probes into the ground to measure the dry density and water content of the soil, it is a time-consuming and destructive procedure. It would therefore be advantageous to develop a new non-destructive TDR system.

Shirley *et al.* [14] first applied a method of measuring the modulus of elasticity by using the bender element to the field of soil mechanics, and presented similar maximum shear moduli of elasticity obtained from the resonant column test and the bender element test. Accordingly, this technique has been employed for a lot of studies (Dyvik *et al.* [15]).

Various instruments have been used to measure the modulus of elasticity which is an indicator of the stress-strain behavior of soil and of the stiffness of the soil. Choi *et al.* [16] applied the DCP (Dynamic Cone Penetrometer) and the LFWD (Light Falling Weight Deflectometer) to the railroad ground, and Park *et al.* [17] assessed the state of railroad subgrade by means of GPR (Ground Penetrating Radar).

The objective of the study was to develop a non-destructive TDR system for measuring the dry density, water content, and modulus of elasticity of soils, without disturbing the ground. Efforts were made to improve the TDR probe design currently commonly used, and to replace it with a flat probe for non-destructive use. The Piezoelectric Stack, which is a type of bender element, was incorporated in the new probe design in order to measure the modulus of elasticity.

## 2. Measuring Methods

### 2.1. Measuring Water Content and Dry Density

The sand cone test and the nuclear gauge test are typically used to measure the dry unit weight in subgrade compaction quality control for roads and railroads. The nuclear gauge test, however, has recently been losing popularity due to the concern about radioactivity. As discussed previously, the TDR technique has been recently introduced.

#### 2.1.1. Sand Cone Test

Civil engineers use the sand cone test very frequently in the field to measure the field soil density and evaluate the soil compaction quality. The degree of soil compaction is generally designated to pass if the measured dry density is larger than 90%–95% of the maximum dry density obtained from the laboratory standard compaction test [18].

**Figure 1:**
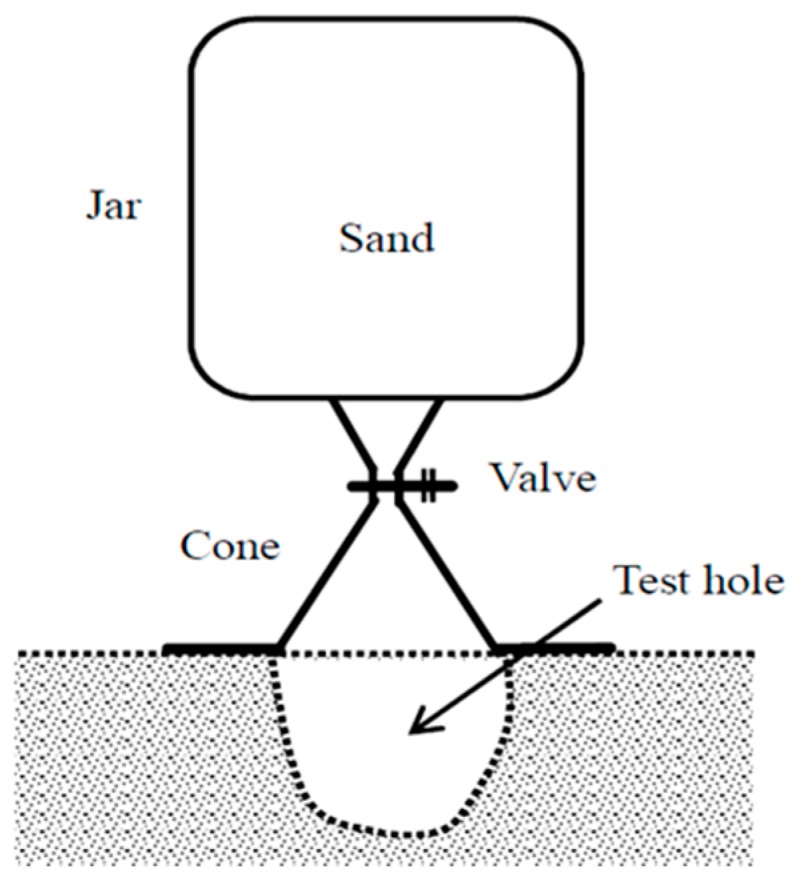
Schematic of field density measurement of compacted soil for sand cone test [17].

Figure 1 presents a schematic of the field density measurement of compacted soil for the sand cone test. The sand cone test may sometimes cause considerable errors depending on the testing personnel and is a destructive testing method. Furthermore, it takes more than one day to obtain the water content with the sand cone test.

#### 2.1.2. TDR Technique

(1) TDR Instrument

**Figure 2:**
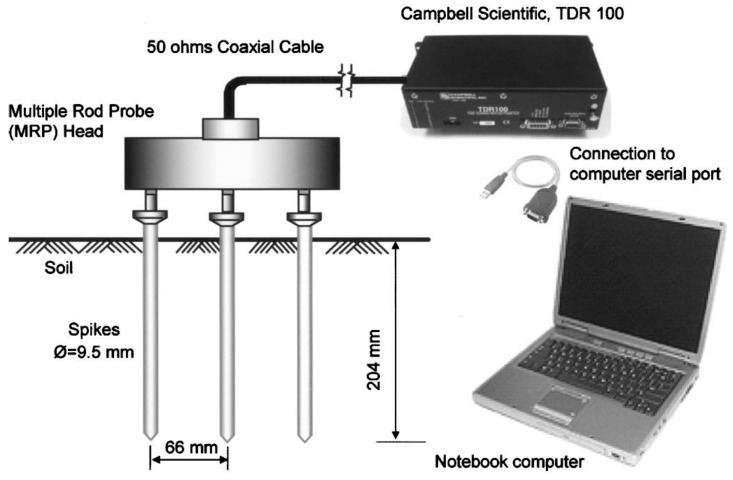
Time Domain Reflectometry (TDR) system components [8].

Figure 2 shows the components of a typical rod type TDR system. The TDR electronic components include a transmission line, power supply, and a data acquisition system. As shown in Figure 2, the transmission line consists of a front panel (Campbell Scientific TDR 100 Pulse generator/sampler (Campbell Scientific Inc., Logan, UT, USA), a 1.8 m-long coaxial cable, a probe head, and a soil probe). The front panel generates a step pulse and the sampler records the reflected signal that is associated with the dielectric properties of the soil being tested. Table 1 shows the main characteristics of the Campbell Scientific device.

The user interface is through the PMTDR program developed by Drnevich *et al.* [20]. The software controls the TDR device and sets vertical and horizontal scales. The sampling time is defined by the length of the record and the number of data points (512, 1024, or 2048), and has to be greater than the time resolution of the equipment presented in Table 1 [21].

(2) Measuring Water Content and Dry Density of Soil by Using the TDR System

**Figure 3:**
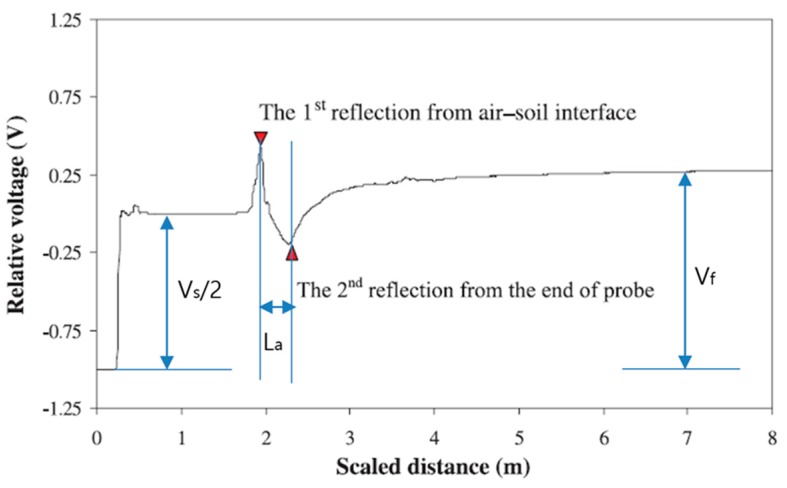
Typical TDR waveform for soil.

In order to obtain the water content and dry density, the TDR system’s rod probes have to be inserted into the ground and then the wave form has to be analyzed as shown in Figure 3. The soil dielectric permittivity (*K_a_*) and electrical conductivity ($EC_{b}$) are important parameters that can be obtained from the TDR waveform (Figure 3). The soil dielectric permittivity and electrical conductivity are determined from Equations (1) and (2).

(1) $$K_{a}={\left(\frac{L_{a}}{L_{p}}\right)}^{2}$$

where, $L_{p}$ is the length of the probe in the soil, and *L_a_* is the distance between reflection from the soil surface and the reflection from the end of the probe.

(2) $$EC_{b}=\frac{1}{C}\left(\frac{V_{s}}{V_{f}}-1\right)$$

where, $V_{s}$ is the source voltage which equals twice the step pulse, *V_f_* is the long term voltage level, *C* is the constant related to probe configuration, and *C* can be derived using Equation (3).

(3) C=2πLpRsln(dodi)

where, $R_{s}$ is the internal resistance of the TDR’s pulse generator, $d_{o}$ and $d_{i}$ are the outer and inner conductor diameters, respectively (Giese and Tiemann, [22]).

Yu *et al.* [8] argued that the calibration equations, represented by means of the volumetric water content, are complex to apply because civil engineers generally use the gravimetric water content in the field. Siddiqui *et al.* [6] suggested a linear calibration of Equation (4) composed of gravimetric water content, which reveals satisfactory results for various soils (Sallam *et al.* [7]).

(4) $$\sqrt{K_{a}}\frac{\rho_{\omega }}{\rho_{d}}=a+b\omega$$

where *a*, *b* are the soil-specific dependent calibration coefficients, $\rho_{\omega }$ is the density of water, $\rho_{d}$ is the dry density of soil, and ω is the gravimetric water content.

Yu *et al.* [8] insisted that the equation does not have a term for density, and the electrical conductivity represented with the volumetric water content is not enough for the application to ground engineering. Yu *et al.* [8] introduced Equation (5) in order to solve a system of two unknown variables, ω and $\rho_{d}$. They suggested Equation (5) on the assumption that the volume of the pore fluid rules the bulk electrical conductivity of the soils because the factor ruling the bulk electrical conductivity ($EC_{b}$) of soils is the electrical conductivity of the pore fluid.

(5) $$\sqrt{EC_{b}}\frac{\rho_{\omega }}{\rho_{d}}=c+d\omega$$

where, $EC_{b}$ is the bulk electrical conductivity of the soils, and *c* and *d* are the soil-specific dependent calibration coefficients.

The TDR signal measured in the field is used to calculate the bulk electric conductivity from the dielectric permittivity calibration Equation (4) and calibration coefficient from the bulk electrical conductivity calibration Equation (5). The dielectric constant, the bulk electrical conductivity and the TDR calibration coefficient are used to calculate the dry density and the water content *in-situ* by using Equations (6) and (7) (ASTM 6780-05 [23]).

(6) $$\rho_{d}=\frac{d\sqrt{K_{a}}-b\sqrt{EC_{b}}}{ad-cb}\rho_{\omega }$$

(7) $$\omega =\frac{c\sqrt{K_{a}}-a\sqrt{EC_{b}}}{b\sqrt{EC_{b}}-d\sqrt{K_{a}}}$$

In summary, even though the TDR system with the rod type probe was developed to improve some shortcomings of the conventional methods (particularly the sand cone test), it requires inserting probes into the ground. This means that it is still a time-consuming and disturbing testing method. Thus, improvement of this TDR system should be sought for quick characterization of the soil.

### 2.2. Measuring Modulus of Elasticity

#### 2.2.1. SASW Method (Spectral Analysis of Surface Waves Method)

**Figure 4:**
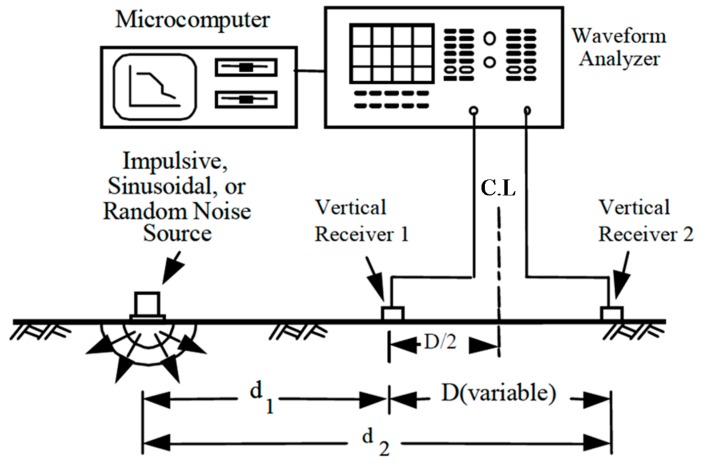
Schematic diagram of Spectral Analysis of Surface Waves Method (SASW) (Stokoe *et al.* [24]).

The surface wave probe technique was designed to address the issue that the surface waves occurring on the surface depend on the vertical distribution of the geological stratum. This technique estimates the shear rigidity of the underground media quickly in the field by measuring surface waves from the impact sources on the ground or structures by means of a plurality of receivers. The SASW is to measure the propagation velocity with the surface to assess the rigidity log in a non-destructive way for the internal media, for example, natural ground, paved systems, and concrete structures. As shown in Figure 4, the process is composed of steps of installing an accelerometer or speedometer at two neighboring points, recording signals propagated along the structure surface or ground surface, converting the signals to the frequency domain and then calculating the propagation velocity of the frequencies related to the surface waves by using the result. The relationship of frequencies determined as such to the surface wave propagation velocity is referred to as surface wave velocity dispersion curves, used to determine the shear wave velocity for each medium depth through inversion analysis.

For the SASW method, several engineers are needed to perform the test, and the dry density and water content cannot be measured. Also, it generally takes considerable time to perform [24].

#### 2.2.2. RPLT (Repetitive Plate Load Test)

Unlike the conventional plate load test, the RPLT uses different maximum loads, increasing the load in each step, the number of loadings, and the time of loading in each step. In particular, loads are repetitively given through load, unload, and reload to remove initial plastic settlement and achieve a fast loading time in each step. Therefore, the RPLT implements faster assessment of compaction quality rigidity in the field than the conventional load test, and removes the great plastic deformation [25].

However, as the same mobilization effort in the RPLT should be made as in the plate load test, this test is also time-consuming. Additionally, the RPLT is only used for measuring the elastic modulus of soil, not for the dry density and the water content.

#### 2.2.3. LFWD (Light Falling Weight Deflectometer)

**Figure 5:**
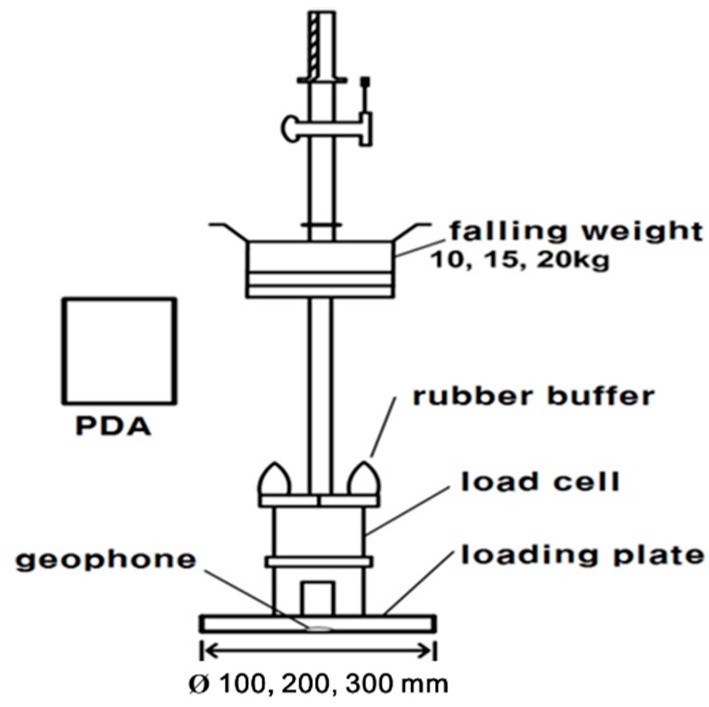
Schematic diagram of the Light Falling Weight Deflectometer (LFWD).

The LFWD, an alternative to the plate load test which is an *in-situ* test, and a portable FWD, was developed in Germany. Generally, the LFWD is composed of a loading device, a loading plate, and a Geophone sensor for measuring settlement in the center. The LFWD is a device for inversely calculating the modulus of elasticity by means of deflections occurring by impacting on the paved surface with a free falling object. It is allowed to control the loading plate size depending on the rigidity of the base ground. The LFWD operates by using deflections measured by means of the sensor in the center of the loading plate to calculate the dynamic deformation coefficient depending on the deflections from the theory of elasticity, on the assumption that the impact force of the falling load is considered as a static load, and the ground is a homogenous elastic body [26]. Figure 5 shows a schematic of the LFWD.

Although the LFWD is useful to measure the elastic modulus of soil, it is not suited for collecting the dry density and the water content.

## 3. Development of a New TDR System

**Figure 6:**
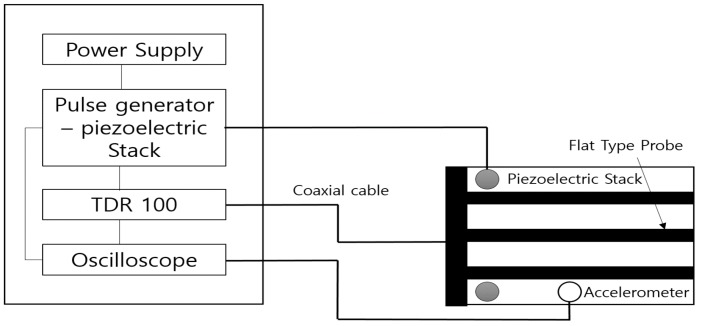
Schematic diagram of the new TDR system.

Figure 6 displays a schematic diagram of the newly developed non-destructive TDR system. As mentioned previously, the new non-destructive TDR system is equipped with a flat-type probe measuring dry density and water content along with the piezoelectric stack measuring the elastic modulus of soil. Since this system weighs about 10 kg, an engineer can carry it easily to perform the field test.

### 3.1. Development of Flat Type Probe

The conventional rod probe TDR disturbs the soil inevitably to some extent because it has to be inserted into the soil to measure the dry density and water content. It would be very difficult to insert the probe into the ground when it encounters gravel and boulders. The conventional TDR test is more advanced than the sand cone test, it still consumes a considerable amount of time to perform the test.

Accordingly, this study aims to develop a flat probe that can measure the dry density and water content on the surface of the soil. As the new TDR system does not disturb the soil, one can measure the dry density and water content of soils rapidly from location to location, leading to significant time and cost savings.

**Figure 7:**
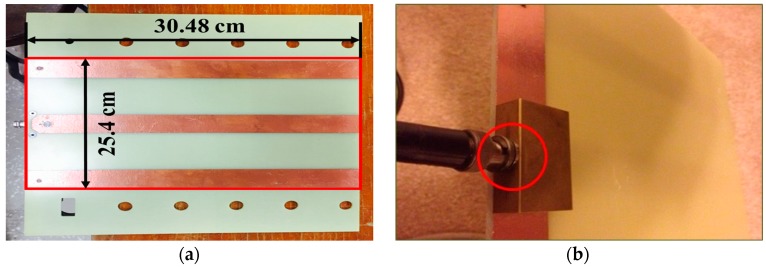
Developed TDR Probe. (**a**) Flat probe; (**b**) connection.

The flat probe is made of copper, and attached to a polymer plate for shielding and diffracting electromagnetic waves. The coaxial cable and the connection are made of stainless steel to avoid corrosion. The probe is shaped for replacing the conventional rod probe. Figure 7 shows the flat-type probe developed in this study.

For an engineer to perform a test conveniently, as shown in Figure 7, the flat-type probe has dimensions of 25.4 cm in width and 30.48 cm in length, and the shielding plate attached to the probe measures 30.48 cm in width and 30.48 cm in length. The dimension of the flat type probe was determined based on the finite element method (FEM) proposed by Knight *et al.* [27] and Ferre *et al.* [28].

Ferré *et al.* [28] first presented a method based on spatial weighting function to numerically determine sample volume of TDR probes. Starting from the point of highest weighting, the summation of the product of weighting function for a small element and its volume is calculated. When the summation is close to a certain percentage, for example 90%, of the summation over the whole calculation domain, the corresponding volume is the sample volume. The calculation of the percentage is shown in Equation (8).

(8) $$f=\frac{100\times \sum_{wh}^{}w_{i}A_{i}}{\iint_{\Omega }w_{i}dA}$$

The spatial weighting function in two dimensions is given by

(9) $$w\left(x,y\right)=|E\left(x,y\right){|}^{2}/\left(\iint_{\Omega }|E_{o}\left(x,y\right){|}^{2}dA\right)$$

The spatial weighting function requires the electrical energy density to be determined, which can be done using finite element method programs.

**Figure 8:**
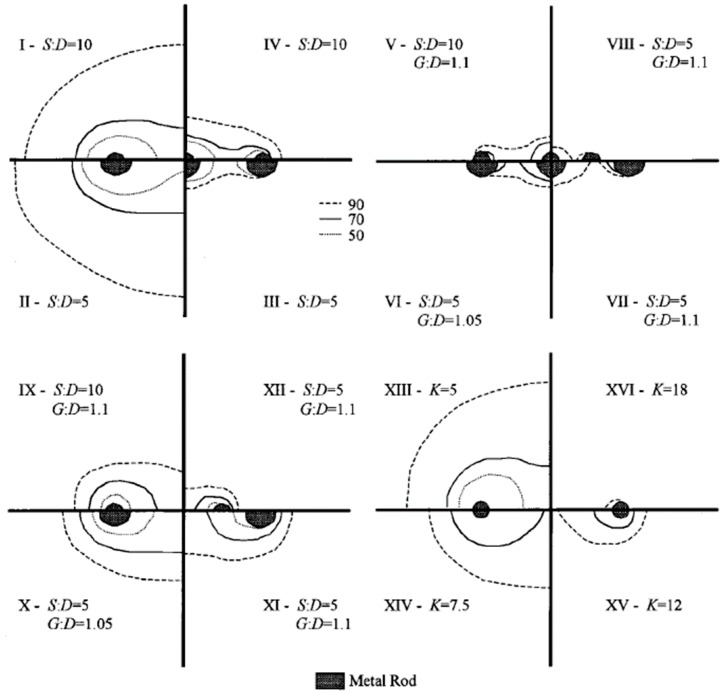
I to IV: percent sample areas of conventional probes; V to XII: coated-rod percent sample areas in a medium with dielectric permittivity 10; and XIII to XVI: coated rods with *S*:*D* = 10, *G*:*D* = 1.1 surrounded by a soil with uniform dielectric permittivity, K, of varying value [28].

In theory, every point in the whole calculation domain has a contribution to the probe response. A percentage of the total contribution should be chosen to characterize the sample volume of the probes. In their study (Ferré *et al.* [28]), 50%, 70%, and 90% were chosen to compare the corresponding areas. Figure 8 shows the calculated sample areas of coated rods. The probe configuration is defined by the separation of the outer rods, *S*, the rod diameter, *D*, and the outer diameter of the rod coatings, *G*. Following the example of Knight *el al.* [27], constant potentials 1 and −1 V were set on the rods of two-rod probes; A constant potential 1 V was set on the center rod of three-rod probes and −1 V was set on the outer rods. The vertical axes and horizontal axes in the figure are lines of symmetry. One subplot represents four probe configurations. The sampling volume of the coated probes is a function of both probe configuration and dielectric permittivity of the coating materials and the medium. More details of the findings can be found in the reference.

**Figure 9:**
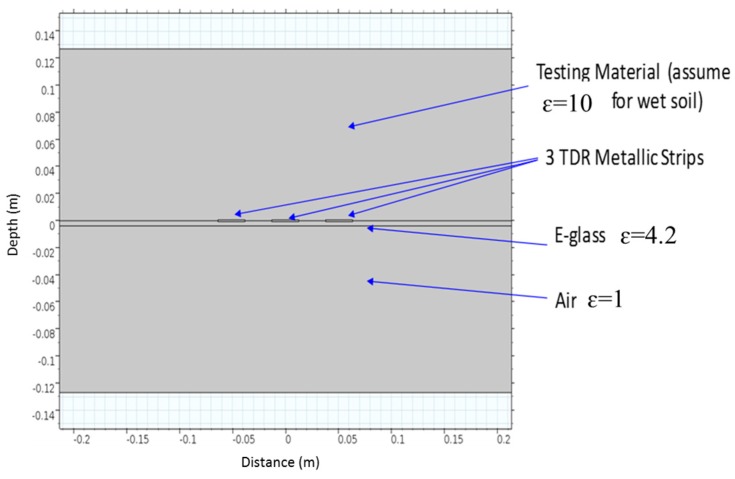
Finite element method (FEM) model.

**Figure 10:**
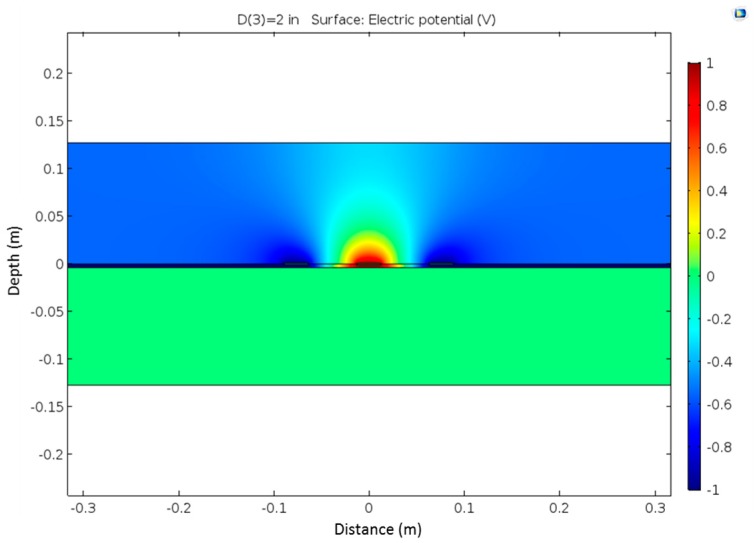
Electric potential distribution.

**Figure 11:**
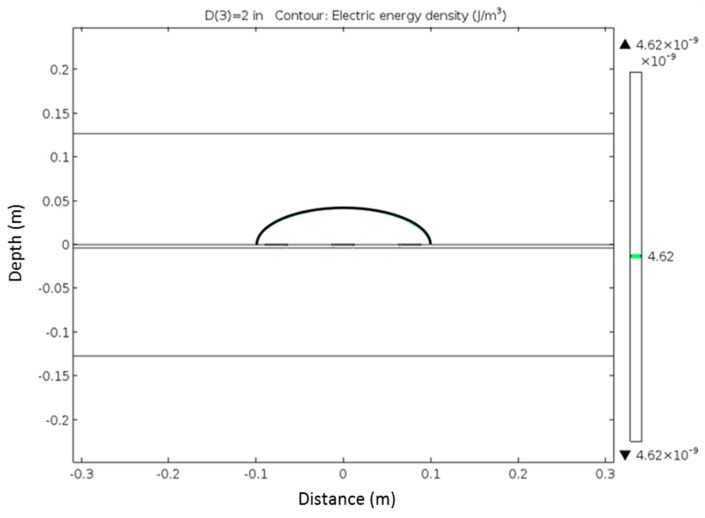
Contour of the electric energy density that demonstrates the effective sampling volume (assuming 90% contribution to the total energy density).

In this design and analysis, 90% was chosen to compare the corresponding volumes. FEM was used to determine the electrical field distribution around the probe. The schematic of the FEM model is shown in Figure 9. The electrical potential distribution is shown in Figure 10. From the electrical field density distribution, the effective sample volume can be determined, which is shown in Figure 11. As in the case of the rod type probes, the center rod serves as the main rod and the outer two rods as the shield rod. The effective sampling volume contributes to 90% of the response to the TDR probe. The effective sampling distance is approximately 7 cm for a permittivity of 10 away from the plate. It is expected that the higher the dielectric permittivity, the higher the sampling volume. Due to the small dielectric permittivity of air, it does not have appreciable influence on the effective sampling volume. The effective sampling volume can be affected by the true dielectric permittivity of the soil being measured. It should be noted that further experimental investigations on sampling volume are required and that this manuscript limits the discussion to the FEM analysis and it application to sandy soils.

Usually the lift thickness for compaction quality is 15 cm. However, to measure the dry density and water content for the 15 cm thick soil, the size of the probe had to be increased five times as the TDR probe developed. Although the longer probe can measure the deeper volume of soil, it is hard for an engineer to carry the TDR system, and it is difficult to have solid contact between the probe and the soil. This is the reason why the current size of the probe was selected.

### 3.2. Combining Flat Type Probe with Piezoelectric Stack

**Figure 12:**
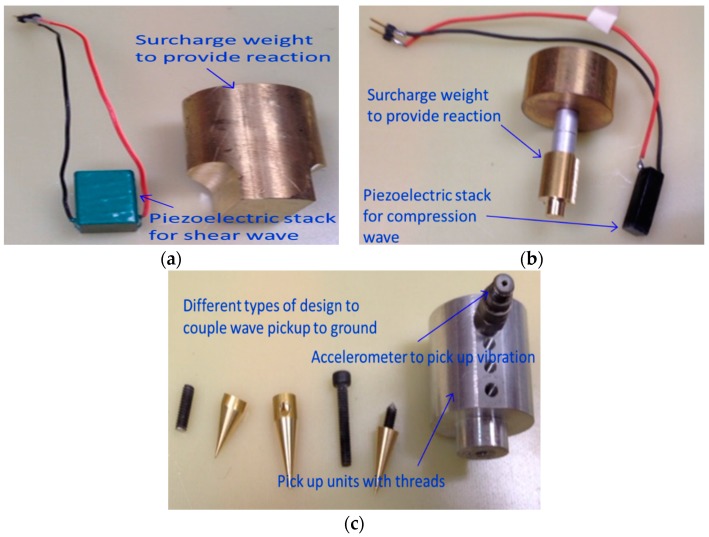
Composition of Piezoelectric Stack. (**a**) Piezoelectric Stack for shear wave and weight; (**b**) Piezoelectric Stack for compression wave and weight; (**c**) Accelerometer to pick up vibration.

The TDR system with the flat type probe is combined with a Piezoelectric Stack, shown in Figure 12, for measuring the modulus of elasticity. Like the SASW, use of the Piezoelectric Stack is based on the principle of measuring the surface wave without penetrating the soil. The original idea was to combine the bender element with the TDR system to measure the modulus of elasticity. However, the Piezoelectric Stack was used because the bender element requires a voltage 10 times larger than the voltage required by the TDR and is not durable to be used on the surface of the ground, but the Piezoelectric Stack requires a voltage smaller than the bender element and is fairly strong. Table 2 illustrates the characteristics of the Piezoelectric Stack.

**Figure 13:**
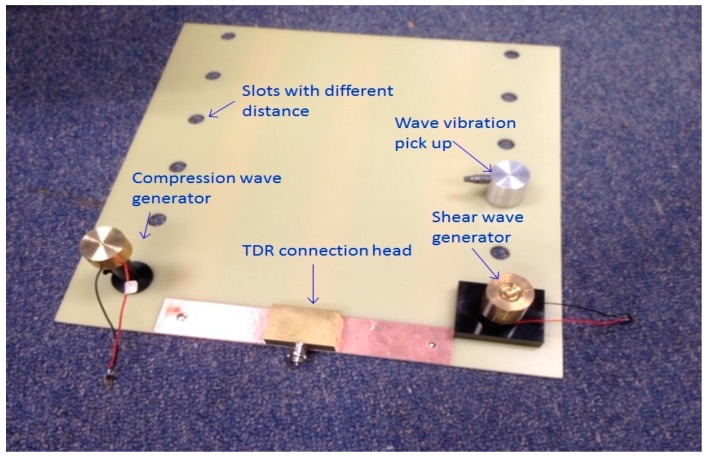
Probe combined with Piezoelectric Stack.

As shown in Figure 13, slots at intervals of about 5 cm were made to measure the modulus of elasticity for various soils by changing the position of the accelerometer which picked up the compression and shear waves, depending on the condition of the measured soils. A weight immediately on the Piezoelectric stack was installed to ensure a solid contact between the Piezoelectric Stack and the ground, resulting in a reliable modulus of elasticity. Figure 12a,b show the piezoelectric stacks and weights, respectively and Figure 12c presents the accelerometer that receives the compression and shear waves. Figure 10 shows the probe combined with the Piezoelectric Stack.

## 4. Discussions of the Test Results

### 4.1. Results of Water Contents and Dry Density

**Figure 14:**
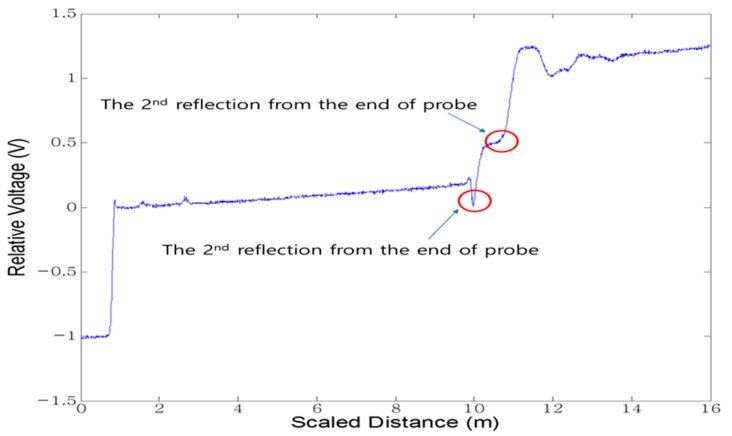
The TDR waveform measured by the flat-type probe.

Figure 14 displays the waveform measured by the flat-type TDR. As shown in Figure 14, the first reflection of the wave occurs at the tip (at the interface point between the air and the soil), while the second reflection occurs at the end of the probe. The dielectric permittivity can be measured using these signals (using the reflection characteristics of electromagnetic wave caused by the change in impedance of the medium around the coaxial cable and the probe), and then the water content and dry density can be obtained.

**Figure 15:**
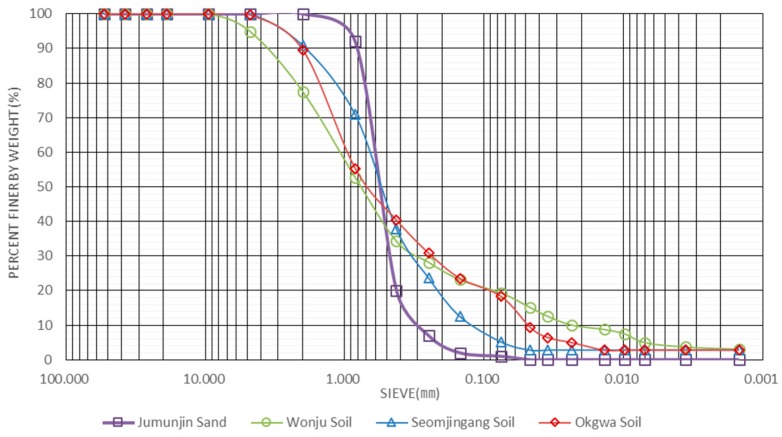
Soil particle size distribution curves.

In order to evaluate its accuracy compared with the standard test, tests with the new TDR system were performed on four different sandy soils that are Jumunjin sand, Wonju soil, Seomjingang soil, Okgwa soil in Korea. Figure 15 and Table 3 show the soil particle size distribution curves and physical properties of the soils used in this study, respectively.

**Figure 16:**
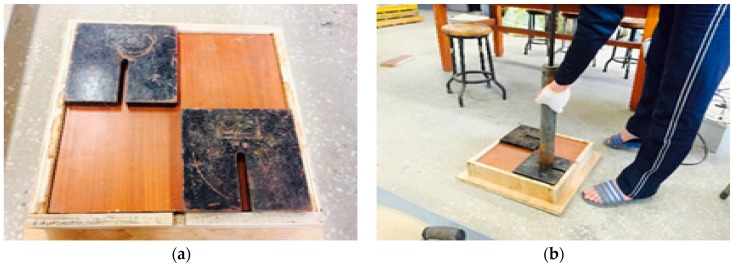
Compaction process. (**a**) Preparation of soil compaction with weight; (**b**) Compaction.

Figure 16 shows the compaction test equipment used in this study. The compaction test was performed using a mold of measurements 35.5 cm × 35.5 cm × 7.5 cm. The soil was placed into the mold in three layers and compacted with the same number of blows per layer (8, 12, and 16 blows for each case) by dropping the rammer on a 4 kg steel plate sitting on top of a wooden plate. The TDR electromagnetic wave was obtained on the surface of the compacted soil after it was flattened and smoothed. For ensuring accuracy of the data, three measurements were done for the same test condition.

**Figure 17:**
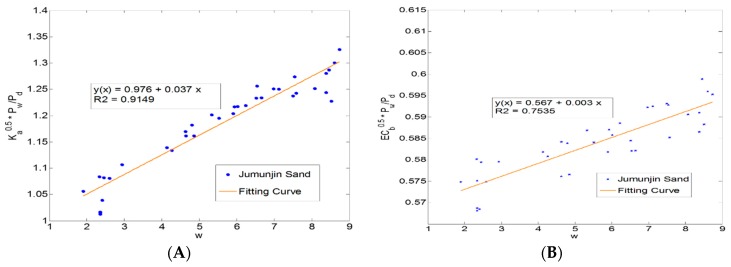
Calibration curve to obtain soil constants for Jumunjin Sand. (**A**) a, b; (**B**) c, d.

The calibration coefficients (a, b, c, and d) of Jumunjun sand are 0.976, 0.037, 0.567, and 0.003, respectively. Figure 17 shows the soil-specific dependent calibration coefficients, a, b, c, and d for Jumunjin sand.

Inserting the soil-specific dependent calibration coefficients, a, b, c and d into Equations (6) and (7) gives the water content and dry density of the soil. The measured water content and dry density with the TDR system were compared with the real values. The moisture content was varied between 0% and 10% and different degrees of compaction were made by changing the number of compaction in each layer.

**Figure 18:**
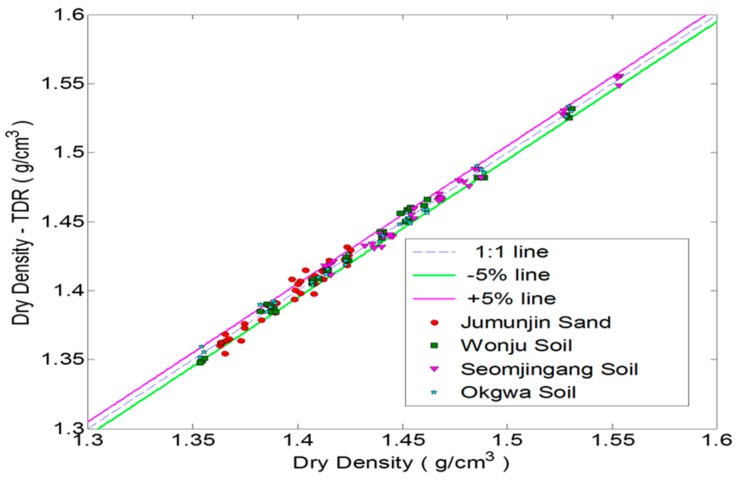
Dry density measured by the flat type TDR and the standard test.

**Figure 19:**
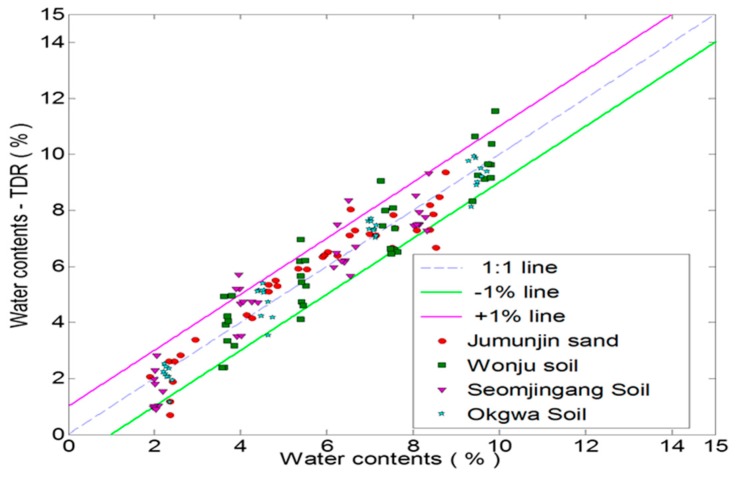
Moisture content measured by flat type TDR and standard test.

Figure 18 and Figure 19 are graphs that compare the moisture content and dry density measured by the new TDR and those measured by the direct measurements of total density and oven-drying water content. The results of the measured and estimated dry density are shown within ±5% deviations from the 1:1 line. Although there are some scatters in the water contents measured by the new TDR system and by the standard test, the new non-destructive TDR system is capable of estimating the moisture content to a reasonable accuracy.

### 4.2. Results of Modulus of Elasticity

In this study, the elastic modulus can be obtained by using the wave speed. The compression and shear wave velocity can be calculated using Equation (10). The compression elastic modulus (*E_p_*) and the shear elastic modulus (*E_s_*) can be obtained using Equations (11) and (12), respectively.

(10) $$V_{s}=V_{p}=D/T_{r}$$

(11) $$E_{p}=\rho {\left(V_{p}\right)}^{2}$$

(12) $$E_{s}=\rho {\left(V_{s}\right)}^{2}$$

where *D* is the distance between the piezoelectric stack and the accelerometer, $T_{r}$ is the travel time for the first direct arrival method, ρ is the soil total density, $V_{s}$ is the shear wave velocity, and $V_{p}$ is the compression wave velocity.

**Figure 20:**
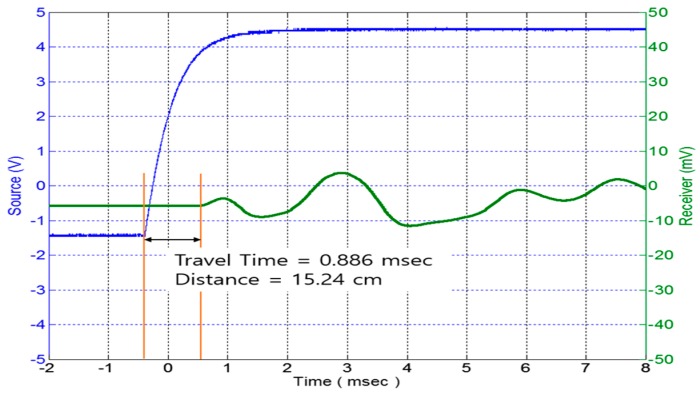
Shear wave speed measured by piezoelectric stack.

**Figure 21:**
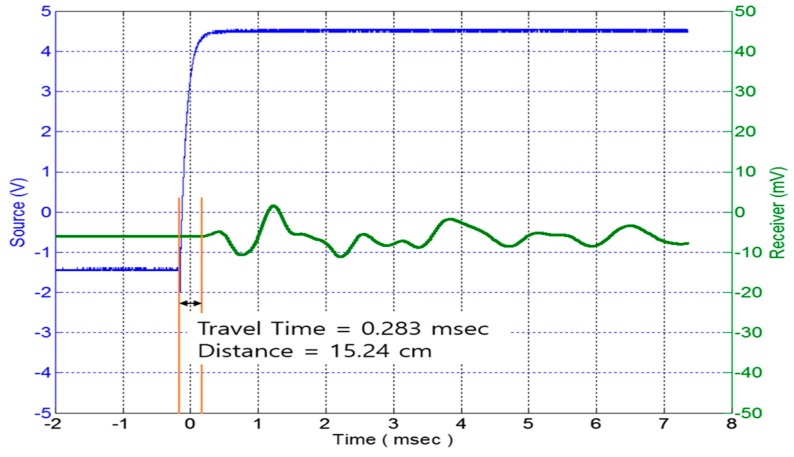
Compression wave speed measured by piezoelectric stack.

In order to assess the capability of the new TDR system that can also measure the elastic modulus, some different types of materials such as asphalt, concrete, and soil were tested. Figure 20 and Figure 21 display both the compression wave and the shear wave measured by the new TDR system, respectively.

Table 4 presents the wave speeds obtained for different materials such as asphalt, concrete, and soil measured by the new TDR system. Note that the modulus of elasticity of the material can be obtained by measuring the compression and shear waves using the Piezoelectric Stack. As can be seen from Table 4, a clear difference in the elastic moduli, calculated using Equation (9), for the three materials is observed. This demonstrates that the new non-destructive TDR system developed can be used as an alternative to conventional methods. For implementation of this non-destructive TDR system in terms of the elastic modulus, creating a correlation between the moduli measured by the new TDR and those by the LFWD will be useful.

## 5. Conclusions

The objective of the study was to develop a new non-destructive TDR system (with a flat type probe combined with a Piezoelectric Stack) that could measure the dry density and water content and elastic modulus of soil, for quick evaluation of soil compaction quality, on the surface of the compacted soil without excavating it. The developed method was tested on four sandy soils with negligible amounts of clay and gravel. The following conclusions can be drawn from the study.

1. A new non-destructive TDR system, capable of measuring the dry density, water content, and elastic modulus has been developed. While the conventional TDR system requires inserting a probe into the ground to measure the soil’s dry density and the water content, the new non-destructive TDR system developed in this study can measure these parameters on the surface of the compacted soil. Based on the test results on standard sand in Korea, the dry density and water content measured by the new TDR system with a flat type probe are in good agreement with those measured by the standard test. This indicates that the new TDR system is feasible and can lead to time and cost savings in measuring dry density and water content.
2. Based on the test results on different types of materials such as asphalt, concrete, and soil, a clear difference in the elastic moduli for the tested materials was observed. This implies that the new TDR system can be used as a tool to measure the stiffness of soil. A further study on developing the correlation between the moduli measured by the new TDR and those by LFWD will be useful.
3. This new non-destructive TDR system is an innovative TDR system that can enable the measurement of moisture content and dry density as well as elastic modulus on the surface of compacted soil. This system will be useful for advancing the compaction quality of soil after it is reinforced with numerous test data on a variety of types of soils. This can be accomplished in further study.

## Figures and Tables

**Table 1:** Characteristics of the Time Domain Reflectometry (TDR) Campbell Scientific device [19].

Campbell Scientific Pulse Generator/Sampler	
Input signal type	Step rise
System rise time	<250 ps
Time resolution	12.2 ps
Bandwidth	1.7 GHz
Output impedance	50 ± 1 ohms
Noise filtering	1 to 128 average
Temperature range	−40 to 55 °C
Dimension	236 mm × 126 mm × 59 mm

**Table 2:** Characteristics of the Piezoelectric Stack [29].

Piezoelectric Stack	
Compressive Strength	8.8 × 10^8^ N/m^2^
Tensile Strength	4.4 × 10^6^ N/m^2^
Young’s Modulus	4.4 × 10^10^ N/m^2^
Poisson’s Ration	0.34
Density	7900 Kg/m^3^
Wires	Red positive, Black negative
Thermal Operation Range	−20 to 80 °C
Thermal Storage Range	−30 to 85 °C
Humidity	<50%

**Table 3:** Physical properties of soils used.

Soil	Classification	Sand (%)	Silt (%)	Clay (%)	Gs	P < No. 200 (%)	Liquid Limit (%)	Plastic Index
Jumunjin Sand	SP	100	0	0	2.65	0	-	-
Wonju Soil	SM	80	17	3	2.58	20	-	-
Seomjingang Soil	SP	95	4	1	2.64	5	-	-
Okgwa Soil	SM	78	16	2	2.60	18	-	-

**Table 4:** Modulus of elasticity measured by the new TDR.

**Asphalt**					
**Wave Type**	**Distance (cm)**	**Signal Start (us)**	**Signal Arrival (us)**	**Wave Speed (m/s)**	**Modulus of Elasticity (MPa)**
Compression Wave	6.5	0	53.01	1226.2	3758.92
Shear Wave	6.5	0	102.02	637.1	1014.74
**Concrete**					
**Wave Type**	**Distance (cm)**	**Signal Start (us)**	**Signal Arrival (us)**	**Wave Speed (m/s)**	**Modulus of Elasticity (MPa)**
Compression Wave	10	0	48.02	2082.5	8673.61
Shear Wave	10	0	128.6	777.6	1209.32
**Soil**					
**Wave Type**	**Distance (cm)**	**Signal Start (us)**	**Signal Arrival (us)**	**Wave Speed (m/s)**	**Modulus of Elasticity (MPa)**
Compression Wave	15	0	283	530.03	421.40
Shear Wave	15	0	886	169.30	42.99

## References

[1] Fellner-Fedegg H. (1969). The Measurement of dielectrics in the time domain. J. Phys. Chem..

[2] Topp G.C., Davis J.L., Annan A.P. (1980). Electromagnetic determination of soil water content: Measurements in coaxial transmission lines. Water Resour. Res..

[3] Roth C.H., Malicki M.A., Plagge R. (1992). Empirical evaluation of the relationship between soil dielectric permittivityand volumetric water content as the basis for calibrating soil moisture measurements by TDR. J. Soil Sci..

[4] Dirksen C., Dasberg S. (1993). Improved calibration of Time Domain Reflectometry soil water content measurements. Soil Sci. Soc. Am. J..

[5] Malicki M.A., Plagge R., Roth C.H. (1996). Improving the calibration of dielectric TDR soil moisture determination taking into account the solid soil. Eur. J. Soil Sci..

[6] Siddiqui S.I., Drnvich V.P. (1995). Use of Time Domain Reflectometry for Determination of Water Content and Density of Soil.

[7] Sallam A.M., White N.K., Ashmawy A.K. (2004). Evaluation of the Purdue TDR Method for Soil Water Content and Density Measurement.

[8] Yu X., Drnevich V.P. (2004). Soil water content and dry density by Time Domain Reflectometry. J. Geotech. Geoenviron. Eng..

[9] Thring L.M., Boddice D., Metje N., Curioni G., Chapman D.N., Pring L. (2014). Factors affecting soil permittivity and proposals to obtain gravimetric water content from time domain reflectometry measurements. Can. Geotech. J..

[10] Ochsner T.E., Horton R., Ren T. (2001). Simultaneous water content, air-filled porosity, and bulk density measurements with thermo-time domain reflectometry. Soil Sci. Soc. Am. J..

[11] Liu X., Ren T., Horton R. (2008). Determination of soil bulk density with thermo-time domain reflectometry sensors. Soil Sci. Soc. Am. J..

[12] Liu X., Lu S., Horton R., Ren T. (2014). *In situ* monitoring of soil bulk density with a thermo-TDR sensor. Soil Sci. Soc. Am. J..

[13] Ren T., Noborio K., Horton R. (1999). Measuring soil water content, electrical conductivity, and thermal properties with a thermo-time domain reflectometry probe. Soil Sci. Soc. Am. J..

[14] Shirley D.J., Hampton L.D. (1978). Shear wave measurements in laboratory sediments. J. Acoust. Soc. Am..

[15] Dyvik R., Madshus C. (1985). Laboratory Measurements of Gmax Using Bender Element.

[16] Choi C., Park C., Lee I., Kim D. Application of *in-situ* testing methods for bearing capacity estimation of railroad roadbed. Proceedings of the Korean Geotechnical Society Conference.

[17] Park J.O., Kim H.K., Jeon I.S. Evaluation of subgrade state in the gyeongbu high speed railway through GPR tests and drilling boreholes. Proceedings of the 2009 Spring Conference of Korean Society for Railway.

[18] Das B.M. (2006). Principles of Geotechnical Engineering.

[19] Campbell Scientific Product Brochures, TDR100 Time-Domain Reflectometer. http://www.campbellsci.com/tdr100.

[20] Drnevich V., Yu X., Lovell J. (2003). Beta Testing Implementation of the Purdue Time Domain Reflectometry (TDR) Method for Soil Water Content and Density Measurement.

[21] Zambrano C.E. (2006). Soil Type Identification Using Time Domain Reflectometry. Master’s Thesis.

[22] Giese K., Tiemann R. (1975). Determination of the complex permittivity from thin-sample time domain reflectometry improved analysis of the step response waveform. Adv. Mol. Relax. Process..

[23] (2005). Standard Test Method for Water Content and Density of Soil in Place by Time Domain Reflectometry (TDR).

[24] Stokoe K.H., Wright S.G., Bay J.A., Roesset J.M. (1994). Characterization of geotechnical sites by SASW method. Tech. Rev. Geophys. Charact. Sites.

[25] Choi C.Y., Lee S.H., Bae J.H., Park D.H. (2011). Evaluation of correlation between Strain modulus (Ev2) and Deformation modulus (E LFWD) using cyclic plate loading test and LFWD. J. Korean Geosynth. Soc..

[26] Kang H.B., Kim K.J., Kang J.T., Kim J.R. (2007). A Study on the estimation of relative compaction on the subgrade using a Portable FWD. J. Korea Inst. Struct. Maint. Insp..

[27] Knight J.H., Ferré P.A., Rudolph D.L., Kachanoski R.G. (1997). A numerical analysis of the effects of coatings and gaps upon relative dielectric permittivity measurement with time domain reflectometry. Water Resour. Res..

[28] Ferré P.A., Knight J.H., Rudolph D.L., Kachanoski R.G. (1998). The sample areas of conventional and alternative time domain reflectometry probes. Water Res. Res..

[29] Piezo System Inc. Large Piezo Stack. http://www.piezo.com/prodstacks1.html.

